# Racz Catheter Technique Versus Conventional Technique in Lumbar Epidural Steroid Injection for Management of Low Back Pain: A Randomized Controlled Trial

**DOI:** 10.5812/aapm-164983

**Published:** 2025-09-07

**Authors:** Ahmed Shehata Abd Elhamid, Mohammed Said ElSharkawy, Khaled Hamama, Ahmed Nada, Amin Elghanam, Doaa Mohamed Ismail, Shaimaa Waheed Zahra

**Affiliations:** 1 Anesthesia and Intensive Care Department, Faculty of Medicine, Port Said University, Port Said, Egypt; 2 Anesthesiology, Surgical Intensive Care and Pain Medicine Department, Faculty of Medicine, Tanta University, Tanta, Egypt; 3 Neurosurgery & Neurointerventional Surgery Department, Faculty of Medicine, Port Said University, Port Said, Egypt; 4 Neurosurgery Department, Faculty of Medicine, Tanta University, Tanta, Egypt; 5 Internal Medicine Department, College of Medicine, Princess Nourah Bint Abdulrahman University, Riyadh, KSA

**Keywords:** Adhesiolysis, Epidural Steroid Injection, Low Back Pain, Oswestry Disability Index, Racz Catheter

## Abstract

**Background:**

Epidural steroid injections (ESIs) are widely employed for managing low back pain (LBP), particularly when conservative treatment fails. The Racz catheter technique offers targeted drug delivery and mechanical adhesiolysis, potentially enhancing outcomes in chronic LBP.

**Objectives:**

The present study aimed to compare the Racz catheter technique and the conventional technique in lumbar epidural steroid injection for the management of LBP.

**Methods:**

This randomized, controlled, double-blind study was conducted on a sample of 60 patients, aged 18 - 65, comprising both sexes, who had persistent lumbar pain, grade 1 spondylolisthesis, and facet osteoarthropathy with small disc findings on radiological examination. Participants were randomized equally into two groups. The Racz catheter group received lumbar epidural steroids using a Racz catheter, while the conventional lumbar group received conventional lumbar steroid injections.

**Results:**

The Visual Analog Scale (VAS) and Oswestry Low Back Disability Questionnaire (OSW) scores were insignificantly different at baseline, immediately post-procedure, and at 1 month between both groups. However, they were significantly lower at 2 months, 4 months, and 6 months in the Racz catheter group compared to the conventional lumbar group (P < 0.05). Incidences of hypotension, paraesthesia, bleeding, and headache were insignificantly different between both groups. Patient satisfaction levels were significantly higher in the Racz catheter group than in the conventional lumbar group (P < 0.05).

**Conclusions:**

The Racz catheter technique is a superior interventional option for lumbar epidural steroid delivery in patients with persistent LBP, providing enhanced pain relief, improved functional outcomes, greater patient satisfaction, and equivalent procedural safety compared to the conventional technique.

## 1. Background

Low back pain (LBP) is often described as pain or discomfort localized between the lower rib margins and the gluteal folds, with or without symptoms extending into the legs (1, 2). Typically, a variety of non-surgical options are recommended to manage lumbosacral radicular pain, including lifestyle modifications, physical therapy, exercise, oral or local analgesics, and epidural steroid injections (ESIs) (3, 4). The primary objective of these conservative strategies is to delay or prevent the necessity for surgery (5, 6). The ESIs are minimally invasive treatments that are extensively used and have been demonstrated to be effective in reducing lumbosacral radicular pain. In addition, they alleviate acute lower back and leg pain by administering corticosteroids into the epidural space through caudal, interlaminar, or transforaminal techniques, frequently in conjunction with local anesthetics (7). Among the most frequently employed interventions for chronic spinal pain are epidural corticosteroid injections. They alleviate pain by reducing inflammatory mediators, reducing vascular permeability, and minimizing C fiber injury (8). The efficacy of local anesthetics administered through epidural injection has been demonstrated in numerous studies, particularly in patients with elevated cerebrospinal fluid protein levels, a marker frequently associated with inflammation (9). Epidural injections continue to be a key tool in pain management, and new technologies have made these procedures even more precise and effective. One such innovation is the Racz catheter epidural adhesiolysis, which helps target medication delivery more accurately and breaks down scar tissue that might be contributing to a patient’s pain (10).

## 2. Objectives

The present study aimed to compare the Racz catheter and conventional techniques in lumbar ESIs for the management of LBP.

## 3. Methods

This randomized, controlled, double-blinded study involved 60 patients (both sexes, aged 18 - 65) who suffered from persistent lumbar pain, grade 1 spondylolisthesis, facet osteoarthritis, and minor disc changes observed through imaging. This study was approved by the institutional ethics committee (NO.: 36264PR800/8/24) and conducted between September 2024 and March 2025. The study procedures followed the guidelines of the World Medical Association (WMA) Declaration of Helsinki, and written informed consent was obtained from all subjects participating in the trial or their next of kin before study commencement. The trial was registered prior to patient enrollment at ClinicalTrials.gov (NCT06599723, principal investigator: Mohammed S. ElSharkawy, Date of registration: 09/19/2024).

Patients were excluded if they were uncooperative, required surgery for lumbar disc prolapse, had a ruptured disc, had contraindications to the procedure such as bleeding disorders or skin infections, or had a contrast medium allergy or a history of prior spine surgery. Before the procedure, all patients underwent a full medical history taking, clinical examination, and laboratory testing. The diagnosis and the level of spinal pathology (either L3-L4 or L5-S1) were confirmed through clinical assessment and imaging (MRI and X-ray), and patients were instructed on how to use a Visual Analog Scale (VAS) to accurately report their pain levels.

### 3.1. Randomization and Blinding

To ensure methodological rigor, patients were randomly assigned using a computer-generated sequence. Each assignment was concealed in a sealed, opaque envelope to maintain allocation secrecy and prevent bias. Participants were split evenly into two groups: One receiving the Racz catheter-assisted lumbar epidural steroid injection, and the other undergoing the conventional technique. Both the patients and the outcome assessors were kept blind to group allocations throughout the study.

For preoperative preparation, all patients had a 20-gauge intravenous cannula inserted and were continuously monitored with pulse oximetry, non-invasive blood pressure readings, and electrocardiography. All patients underwent standardized preoperative preparation, which included the insertion of a 20-gauge (G) peripheral intravenous cannula to facilitate vascular access. Continuous monitoring was implemented using pulse oximetry, non-invasive blood pressure monitoring, and electrocardiography. All participants received a prophylactic intravenous dose of 1 gram of cefazolin prior to the procedure (11).

### 3.2. Conventional Lumbar Epidural Technique

**Figure 1.**
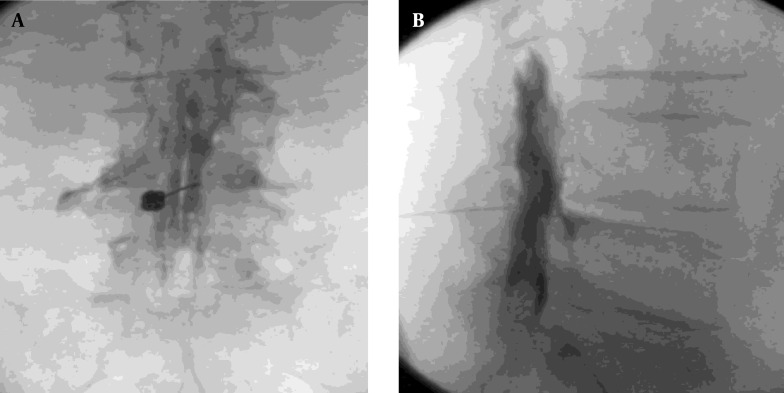
Epidural A, AP view; and B, lateral view after contrast administration

In the prone position, fluoroscopic guidance was established using a C-arm positioned anteroposteriorly. The target intervertebral level was identified by localizing the midpoint of the corresponding disc space. A 25-G short needle was used to infiltrate the skin and subcutaneous tissues with a local anesthetic solution, advancing to the laminar surface. A small window beneath the posterior lumbar spinous process was examined to determine the most favorable needle trajectory. To optimize patient comfort, a route was selected that circumvented the periosteum and minimized traversal through posterior paraspinal musculature. Following anatomical localization, the entry point was marked adjacent to the midline. The skin was sterilized, draped, and anesthetized with 2 mL of 2% lidocaine. A 16- or 18-gauge Touhy needle from the B-BRAUN Perifix^®^ Filter set was introduced through the anesthetized tract. Fluoroscopic imaging was intermittently employed to assess needle depth and angulation as it was advanced toward the dorsal surface of the ligamentum flavum. Upon reaching this landmark, the stylet was withdrawn and connected to extension tubing filled with 10 mL of sterile, non-bacteriostatic saline. The epidural space was accessed using the loss-of-resistance technique. Subsequently, 2 mL of a diluted contrast medium (Omnipaque^©^, GE Healthcare, Shanghai, China) was administered to verify correct placement. A confirmatory fluoroscopic image was obtained to demonstrate the appropriate epidural spread of the contrast agent, ensuring accurate needle positioning and ruling out inadvertent intrathecal injection. The contrast medium was clearly visualized at the needle tip, typically tracking anterior to the ligamentum flavum. In ideal placements, the contrast distribution appeared linear, although a globular pattern could occasionally be seen at the injection site. When ambiguity regarding epidural localization arose, additional fluoroscopic images at adjacent levels were acquired to confirm accurate placement. Once needle positioning was conclusively verified, a therapeutic solution was administered, consisting of 80 mg (2 mL) triamcinolone acetonide, 4 mL of 0.5% bupivacaine, 2 mL of 2% lidocaine, and 4 mL of normal saline. The needle was then carefully withdrawn, concluding the procedure (Figure 1). 

### 3.3. Racz Catheter Technique

**Figure 2.**
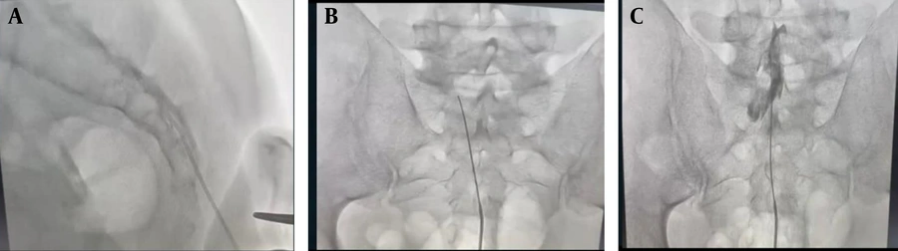
A, entry of Racz catheter; B, Recz catheter in epidural space; and C, Racz catheter after the injection of contrast

In the operating room, the patient was positioned prone with a pillow placed beneath the abdomen to reduce lumbar lordosis and another under the ankles to enhance comfort. Following sterile preparation, the sacral hiatus was identified either by palpation, just caudal to the sacral cornua, or under fluoroscopic guidance. Local anesthesia was administered subcutaneously at a point approximately one inch lateral and two inches caudal to the sacral hiatus. A 16-G Racz needle (Epimed International, Inc., Johnstown, NY) was introduced at a 45° angle under fluoroscopic guidance. Upon traversing the sacral hiatus, the needle angle was adjusted to approximately 30°, and appropriate entry into the epidural space was confirmed. After negative aspiration, 2 mL of iodinated contrast was injected to verify epidural placement by observing the contrast distribution fluoroscopically. The bevel of the needle was then rotated ventrolaterally, and an 18-gauge Racz catheter, pre-bent at the tip to 2.5 cm, was advanced at a 30° angle into the epidural space. Under continuous anteroposterior fluoroscopic monitoring, the catheter tip was navigated toward the ventrolateral epidural space at the target spinal level. Once in position, an additional 2 - 3 mL of contrast agent was administered to confirm proximity to the affected nerve root. After proper catheter placement was verified, a therapeutic mixture consisting of 80 mg (2 mL) triamcinolone acetonide, 4 mL of 0.5% bupivacaine, 2 mL of 2% lidocaine, and 4 mL of normal saline was slowly injected. The catheter was then carefully withdrawn, completing the intervention (Figure 2). 

Patients in whom catheter advancement was impeded due to dense epidural adhesions were excluded from the study.

### 3.4. Postoperative Care

Postoperative care included a structured pharmacological regimen for pain management and infection prevention. This comprised oral antibiotics for five days, intramuscular diclofenac sodium (75 mg every 12 hours for one week) for analgesia, gastric protectants (omeprazole 20 mg orally, every 12 hours for one week) to prevent NSAID-induced gastric irritation, and muscle relaxants (baclofen 10 mg orally, every 12 hours) to alleviate muscle spasms. A multimodal analgesic protocol was implemented, including paracetamol (1 g every 6 hours) for baseline pain control. Rescue analgesia with morphine was administered as a 3 mg bolus when the VAS score exceeded 3, with additional doses given every 30 minutes as needed until the VAS score fell below 4. The VAS assessments were conducted preoperatively, immediately postoperatively, and at 1-, 2-, 4-, and 6-month post-procedure (12).

To assess functional disability, the Oswestry Low Back Disability Questionnaire (OSW) was utilized. This validated instrument evaluates functional impairment associated with LBP through 10 items, each scored from 0 to 4, with total scores categorized as follows: 0 - 4 (no disability), 5 - 14 (mild disability), 15 - 24 (moderate disability), 25 - 34 (severe disability), and 35 - 50 (complete disability). The questionnaire was administered preoperatively to establish a baseline, and then reassessed at 1, 2, 4, and 6 months after the procedure (13).

We gauged patient satisfaction using a 5-point Likert scale, where scores ranged from 1 (extremely dissatisfied) to 5 (extremely satisfied) (14). We documented any complications that occurred during or after the procedures. The primary outcome of the study was the score from the OSW Questionnaire, used to evaluate functional improvement and reduction in disability. Secondary outcomes included the severity of pain measured by VAS scores, patient satisfaction ratings, procedure duration, and the rate of procedural complications.

### 3.5. Sample Size Calculation

For the sample size calculation, we used G*Power software version 3.1.9.2 (University of Kiel, Germany). To ensure accurate planning, a preliminary pilot study was conducted involving five patients in each group. Based on the pilot results, the mean Oswestry Disability Index (ODI) score for patients receiving the conventional lumbar epidural technique was 24.60 ± 13.67, whereas those treated with the Racz catheter technique had a mean ODI score of 13.40 ± 7.70. The required sample size was calculated based on an effect size of 1.009, a confidence level of 95%, and a study power of 90%. An equal allocation ratio (1:1) between groups was maintained to ensure balanced comparisons. To account for potential dropouts or losses to follow-up, an additional eight participants were added to each group. Therefore, the final required sample size was set at 30 patients per group.

### 3.6. Statistical Analysis

The statistical evaluation of the collected data was performed using SPSS software, version 27 (IBM^©^, Armonk, NY, USA). To determine whether the data followed a normal distribution, the Shapiro-Wilk test was applied, complemented by histogram visualization for graphical assessment. For parametric quantitative variables, data were expressed as mean ± standard deviation (SD) and analyzed using an unpaired Student’s *t*-test to compare differences between groups. In contrast, non-parametric quantitative variables were represented as median and interquartile range (IQR) and assessed using the Mann-Whitney test to account for data that did not meet normality assumptions. Categorical data were summarized as frequencies and percentages and evaluated using either the chi-square test or Fisher’s exact test, depending on the expected frequency distribution. A two-tailed statistical approach was employed to ensure rigorous analysis, with a significance threshold set at P < 0.05.

## 4. Results

**Figure 3.**
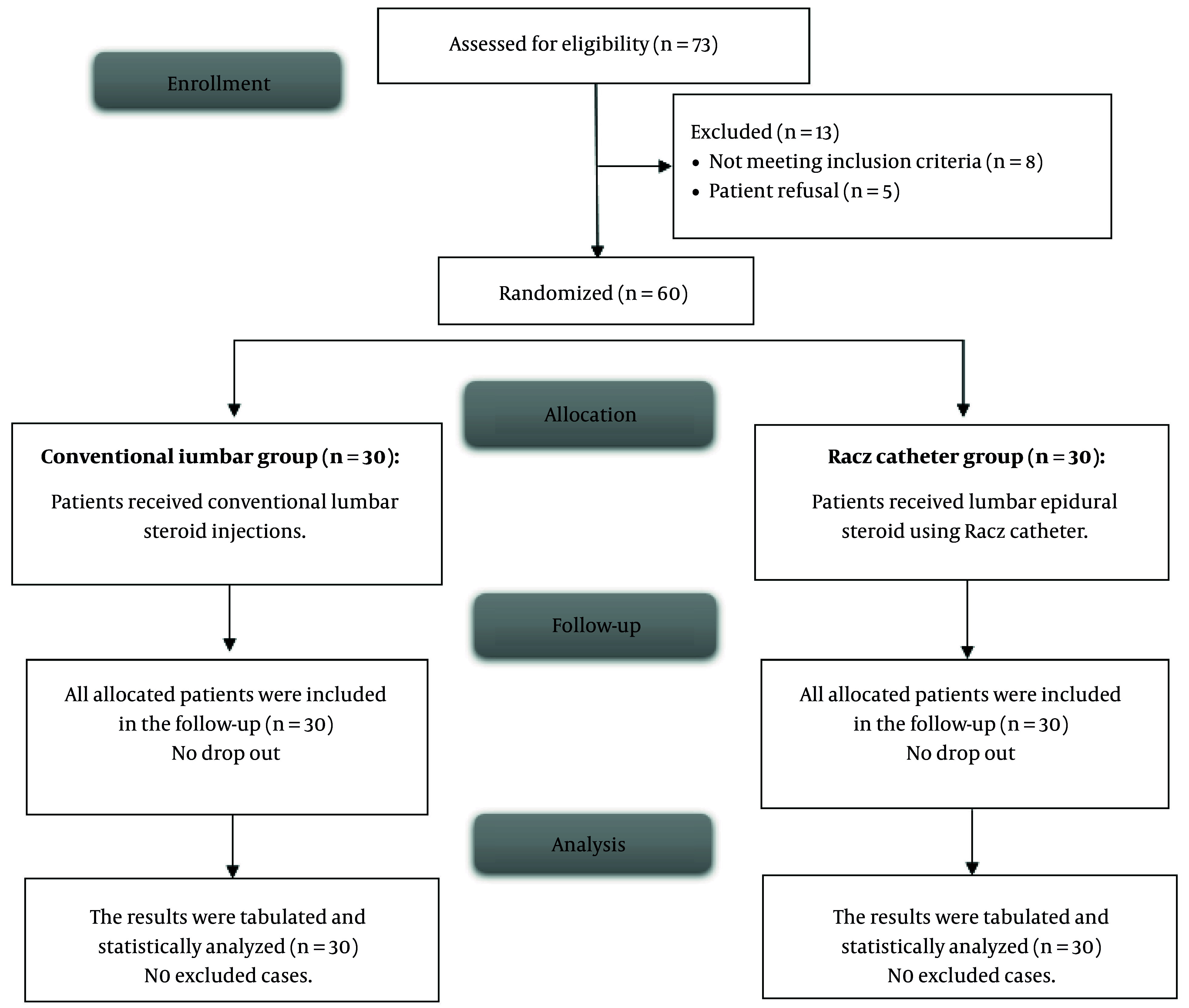
CONSORT flow chart of the enrolled patients

Figure 3 illustrated that 73 cases were enrolled, and their eligibility for participation was assessed. Eight patients did not meet the inclusion criteria, and five refused to participate, resulting in a total of 60 cases who were randomized into two groups for subsequent analysis.

Table 1 indicated that demographic data and procedure time were similar across the cohorts.

**Table 1.** Demographic Data and Procedure Time of the Studied Groups ^a^

Variables	Conventional Lumbar Group (n = 30)	Racz Catheter Group (n = 30)	P-Value
**Age (y)**	38.93 ± 11.92	41.53 ± 10.78	0.379
**Sex**			0.176
Male	22 (73.33)	17 (56.67)	
Female	8 (26.67)	13 (43.33)	
**Weight (kg)**	78.63 ± 5.8	77.63 ± 4.87	0.473
**Height (cm)**	167.43 ± 6.72	167.7 ± 6.41	0.876
**BMI (kg/m** ^**2**^ **)**	28.07 ± 1.34	27.62 ± 1.35	0.204
**ASA physical status**			0.559
I	23 (76.67)	21 (70)	
II	7 (23.33)	9 (30)	
**Procedure time (min)**	23.17 ± 3.59	24.83 ± 3.34	0.068

Abbreviations: BMI, Body Mass Index, ASA, American Society of Anesthesiologists.

^a^ Data are presented as No. (%) or mean ± SD.

Table 2 showed that VAS and OSW scores were insignificantly different at baseline, immediately post-procedure, and at 1 month between both groups. However, they were significantly lower at 2 months, 4 months, and 6 months in the Racz catheter group compared to the conventional lumbar group (P < 0.05).

**Table 2.** Visual Analogue Scale and Oswestry Low Back Disability Questionnaire of the Studied Groups ^a^

Variables	Conventional Lumbar Group (n = 30)	Racz Catheter Group (n = 30)	P-Value
**VAS (mo)**			
Baseline	7 (6.25 - 8)	6 (5.25 - 8)	0.124
Immediate post-procedure	5 (4 - 6)	4 (3.25 - 5)	0.069
1	7 (6.25 - 8)	6 (5.25 - 8)	0.074
2	5 (4 - 6)	4 (3.25 - 5)	0.006 ^b^
4	2 (2 - 4)	1 (1 - 3)	0.019 ^b^
6	2 (1 - 3)	1 (1 - 1.75)	0.002 ^b^
**OSW (mo)**			
Baseline	18.77 ± 7.77	21.5 ± 6.04	0.134
Immediate post-procedure	15.87 ± 7.62	14.7 ± 6.1	0.515
1	12.57 ± 7.25	9.8 ± 5.9	0.111
2	9.27 ± 7.15	5.33 ± 4.52	0.014 ^b^
4	7.5 ± 6.9	3.77 ± 3.37	0.010 ^b^
6	5.93 ± 5.72	2.67 ± 2.59	0.006 ^b^

Abbreviations: VAS, Visual Analog Scale, OSW, Oswestry Low Back Disability Questionnaire.

^a^ Data are presented as mean ± SD or median (IQR).

^b^ A P ≤ 0.05 is considered statistically significant.

Table 3 indicated that incidences of hypotension, paresthesia, bleeding, and headache were insignificantly different between both groups. There were no occurrences of bending of the needle tip, shearing of the catheter, migration of the catheter, misplacement of the catheter, blocking of the catheter, or infection in any patient in both groups. Patient satisfaction levels were significantly higher in the Racz catheter group than in the conventional lumbar group (P < 0.05).

**Table 3.** Side Effects and Patients’ Satisfaction of the Studied Groups ^a^

Variables	Conventional Lumbar Group (n = 30)	Racz Catheter Group (n = 30)	P-Value
**Side effects**			
Bending of the tip of the needle	0 (0)	0 (0)	-
Shearing of the catheter	0 (0)	0 (0)	-
Misplacement of the catheter	0 (0)	0 (0)	-
Blocking of the catheter	0 (0)	0 (0)	-
Bleeding	1 (3.33)	0 (0)	1
Hypotension	2 (6.67)	1 (3.33)	1
Migration of the catheter	0 (0)	0 (0)	-
Paresthesia	1 (3.33)	0 (0)	1
Headache	1 (3.33)	0 (0)	1
Infection	0 (0)	0 (0)	-
**Patients’ satisfaction**			0.021 ^b^
Extremely satisfied	8 (26.67)	19 (63.33)	
Satisfied	9 (30)	7 (23.33)	
Neutral	11 (36.67)	3 (10)	
Unsatisfied	2 (6.67)	1 (3.33)	
Extremely dissatisfied	0 (0)	0 (0)	

^a^ Data are presented as No. (%).

^b^ A P ≤ 0.05 is considered statistically significant.

## 5. Discussion

The approaches employed in chronic lumbar radicular pain management encompass pharmacological analgesics, centrally acting agents, lumbar epidural and transforaminal ESIs, physical therapy and rehabilitation, dorsal root ganglion pulse radiofrequency, and epidural lysis (15). Several investigations have indicated that epidural neuroplasty may cure chronic LBP (16-18). Percutaneous epidural neuroplasty (PEN) is often used in patients with chronic LBP or those who have not improved after back surgery syndrome (19).

This study found a significantly lower OSW (at 2, 4, and 6 months) in the Racz group than in the conventional lumbar group (9.27 ± 7.15 vs. 5.33 ± 4.52, 7.5 ± 6.9 vs. 3.77 ± 3.37, and 5.93 ± 5.72 vs. 2.67 ± 2.59, respectively). In agreement with our findings, Ege (20) found significant decreases in ODI scores one and six months after epidural neuroplasty using the Racz catheter, confirming our findings (P < 0.001). Also, Choi et al. (18) compared 6-month outcomes of endoscopic epidural neuroplasty and PEN in lower back pain. They noted that ODI scores (at 1 and 6 months) decreased significantly compared to pre-PEN (31.7 ± 3.6, 27.8 ± 1.8, and 26.9 ± 2.3, respectively).

Postoperative pain (at 2, 4, and 6 months) was significantly improved in the Racz cohort compared to the conventional lumbar cohort. Similarly, Ege (20) found that epidural neuroplasty employing the Racz catheter resulted in considerably lower pain levels after one and six months compared to before treatment (P < 0.001). Also, Choi et al. (18) showed that pain scores (at 1 and 6 months) decreased significantly compared to pre-PEN (6.5 ± 0.8, 2.3 ± 0.7, 4.6 ± 1.0, and 4.3 ± 0.7, respectively).

Additionally, Moon et al. (21) evaluated cervical epidural neuroplasty utilizing a Racz catheter, clinical outcomes, and identified predictive factors for its efficacy in patients with cervical spinal pain. They exhibited that pain scores (at 1, 3, 6, and 12 months) decreased significantly compared to baseline. The safety profile of both techniques was comparable, with similar incidences of side effects (hypotension, paresthesia, headache, and infection). Also, patients had similar satisfaction rates in both the Racz and conventional lumbar groups.

The comparable safety profile between epidural neuroplasty using a Racz catheter and classic lumbar fixation alone can be attributed to several factors. Both procedures are considered minimally invasive compared to open surgical techniques. While lumbar fixation involves surgical stabilization of the spine, epidural neuroplasty using a Racz catheter is typically performed percutaneously. This reduces overall risk factors associated with more invasive surgeries (22).

The research is restricted by the relatively small sample size, single-center settings, and short-term follow-up (6 months). Larger-scale studies could help confirm these findings and provide a more robust assessment of both the efficacy and safety of the Racz catheter technique in various patient subgroups with different grades of spondylolisthesis.

### 5.1. Conclusions

The Racz catheter technique is a superior interventional option for lumbar epidural steroid delivery in patients with persistent LBP. It provides enhanced pain relief, improved functional outcomes, greater patient satisfaction, and equivalent procedural safety compared to the conventional technique.

## Data Availability

The dataset presented in the study is available on request from the corresponding author during submission or after publication.

## Clinical Trial Registration Code:

NCT06599723.
